# Meta-analysis on the prevalence of REM sleep behavior disorder symptoms in Parkinson’s disease

**DOI:** 10.1186/s12883-017-0795-4

**Published:** 2017-02-04

**Authors:** Jia Zhang, Chuan-Ying Xu, Jun Liu

**Affiliations:** Department of Neurology & Institute of Neurology, Ruijin Hospital affiliated to Shanghai Jiaotong University School of Medicine, Shanghai, People’s Republic of China

**Keywords:** Parkinson’s disease, RBD symptoms, Non-motor symptoms, Meta-analysis, Prevalence

## Abstract

**Background:**

Our study was aimed to evaluate the risk of a selected non-motor symptom, namely rapid eye movement behavior disorder (RBD) symptoms, among patients with newly diagnosed Parkinson disease compared with health controls.

**Methods:**

The Preferred Reporting Items for Systematic Reviews and Meta-Analyses (PRISMA) guidelines for meta-analysis and Cochrane manual were followed. Studies on RBD symptoms and PD were searched using PubMed, Embase, Web of Science and Cochrane library databases. All studies were published before August 3^rd^, 2016. Eligible studies were those that reported a prevalence of RBD symptoms among newly diagnosed PD and health control. Pooled odds ratios (ORs) with 95% confidence intervals (CIs) were calculated by random-effected models. Heterogeneity across studies was assessed using Cochran Q and I^2^ statistics.

**Results:**

We identified eight studies including 2462 PD patients and 3818 health controls. The overall prevalence of RBD symptoms in PD was 582/2462 (23.6%) compared to 131/3818 (3.4%) in control. And the pooled OR was 5.69 (95% CI 3.60 to 9.00; *p* = 0.001) with a moderate heterogeneity I^2^ = 70.5%. After excluding the study of low weight, the overall polled OR was 3.54 (95% CI 2.77 to 4.52; *p* < 0.00001) and the heterogeneity was completely eliminated (I^2^ = 0%).

**Conclusions:**

RBD symptoms are common non-motor symptoms of PD, and people with PD are at a higher risk of developing RBD. Further studies are needed to understand the natural history of RBD symptoms in PD and its etiological and clinical implications.

## Background

Parkinson’s disease (PD) is the second most prevalent neurodegenerative disease with a prevalence of approximately 0.4% in people aged 65 and older, and it increases with age [1]. Apart from motor dysfunction, PD patients also suffer from a variety of non-motor symptoms (NMS), such as hyposmia, rapid eye movement sleep behavior disorder (RBD), constipation and depression [2]. However, certain non-motor symptoms might precede the development of motor symptoms by many years [3]. RBD is characterized by the loss of normal atonia of REM sleep, resulting in an apparent acting out of dream content [4]. It has been reported that RBD as a premotor feature occurred in 38% of patients who developed PD in a mean interval of 3.5 years after a RBD diagnosis [5]. The presence of RBD in prodromal PD is consistent with the Braak hypothesis which implies that it may occur as the result of underlying Lewy pathogenesis at lower brainstem before invading substantial nigra [6]. However, reported prevalence of RBD in PD patients varies greatly from 20 to 72% [7], and the prevalence of probable RBD was estimated to be about 4.9% in the general population [8]. To date, no meta-analysis has examined the magnitude of risk associated with PD and the development of RBD. We therefore conducted a meta-analysis to estimate the prevalence of RBD symptoms among patients with newly diagnosed PD compared with health controls.

## Methods

### Search strategy

We conducted a systematic search of original published studies that reported the prevalence of RBD symptoms in PD and health controls. Two researchers (JZ and CYX) independently undertook a search on PubMed, Web of Science, Embase and Cochrane library databases for literature published in English, which were published before August 3^rd^, 2016. We combined medical subject heading (MeSH) terms including “PD, RBD” and text terms including “Idiopathic Parkinson's Disease, Lewy Body Parkinson Disease, Primary Parkinsonism, Parkinsonism, Primary Parkinson Disease, Paralysis Agitans” and “REM Behavior Disorder, Rapid Eye Movement Sleep Behavior Disorder” as our search strategy. Restrictions were made to observational epidemiologic studies involving humans. Titles and abstracts were screened for their suitability. Articles whose abstracts did not report on RBD symptoms and PD were excluded. Full articles were then obtained and reviewed to determine suitability for inclusion or exclusion. Differences of opinion between the researchers were resolved through discussion. The reference lists of all full articles were included. In addition, references from reviews that were identified in the original study, were hand-searched as additional articles, and then subjected to the same filtering process as described above.

### Inclusion criteria

Published articles that met the following criteria were included: 1) observational studies with a cohort, case-control or cross-section design; 2) cases where patients were diagnosed with PD according to standard clinical criteria, such as UK Parkinson’s Disease Society Brain Bank Criteria [9]; 3) controls were drawn from the same population as cases; 4) controls were healthy or had no history of neurological disease; 5) RBD symptoms in controls were assessed over the same time period as patients; 6) RBD symptoms were assessed by means of polysomnography (PSG), or structured questionnaire, or coded in patient medical records or medication used to treat RBD symptoms and 7) original data were reported.

### Exclusion criteria

We excluded studies for the following reasons: 1) studies in the form of case report, review, conference abstracts, or letters that did not report new data; 2) studies that only provided risk estimates such as relative risk without numbers of individuals; 3) duplicate populations; 4) did not provide adequate details of the control group; 5) did not report sufficient data to calculate risk estimates; and 6) non-English publications or non-human studies.

### Data extraction

Two researchers (JZ and CYX) reviewed all abstracts independently either to determine the eligibility criteria or for examining the appropriateness on the research issue. Data were compared and disagreements were resolved by a consensus. The following information was extracted from each study, and included: name of first author, year of publication, study data, study design, sample size, patient mean age, geographical region, RBD symptoms assessment method and other relevant study characteristics. Finally, studies were assessed for quality using the Newcastle-Ottawa Scale (NOS) [10].

### Statistical analysis

Measures of effect were combined using standard meta-analysis methods. We used the odds ratio (OR) with a 95% confidence interval (CI) as the metric of risk, using the random effects model. Heterogeneity across studies was evaluated by the Cochrane’s Q statistic and by I^2^ test, the percentage of I^2^ around 25, 50 and 75% mean low, medium and high heterogeneity [11]. A sensitivity analysis was conducted to evaluate the source of heterogeneity. Publication bias was assessed using the Egger test and a funnel plot [12]. Statistical analysis was undertaken in STATA (version 12.0; StataCorp, Corporation, College Station, TX, USA) and RevMan (version 5.1; the Cochrane Collaboration, Oxford, United Kingdom).

## Results

**Fig. 1:**
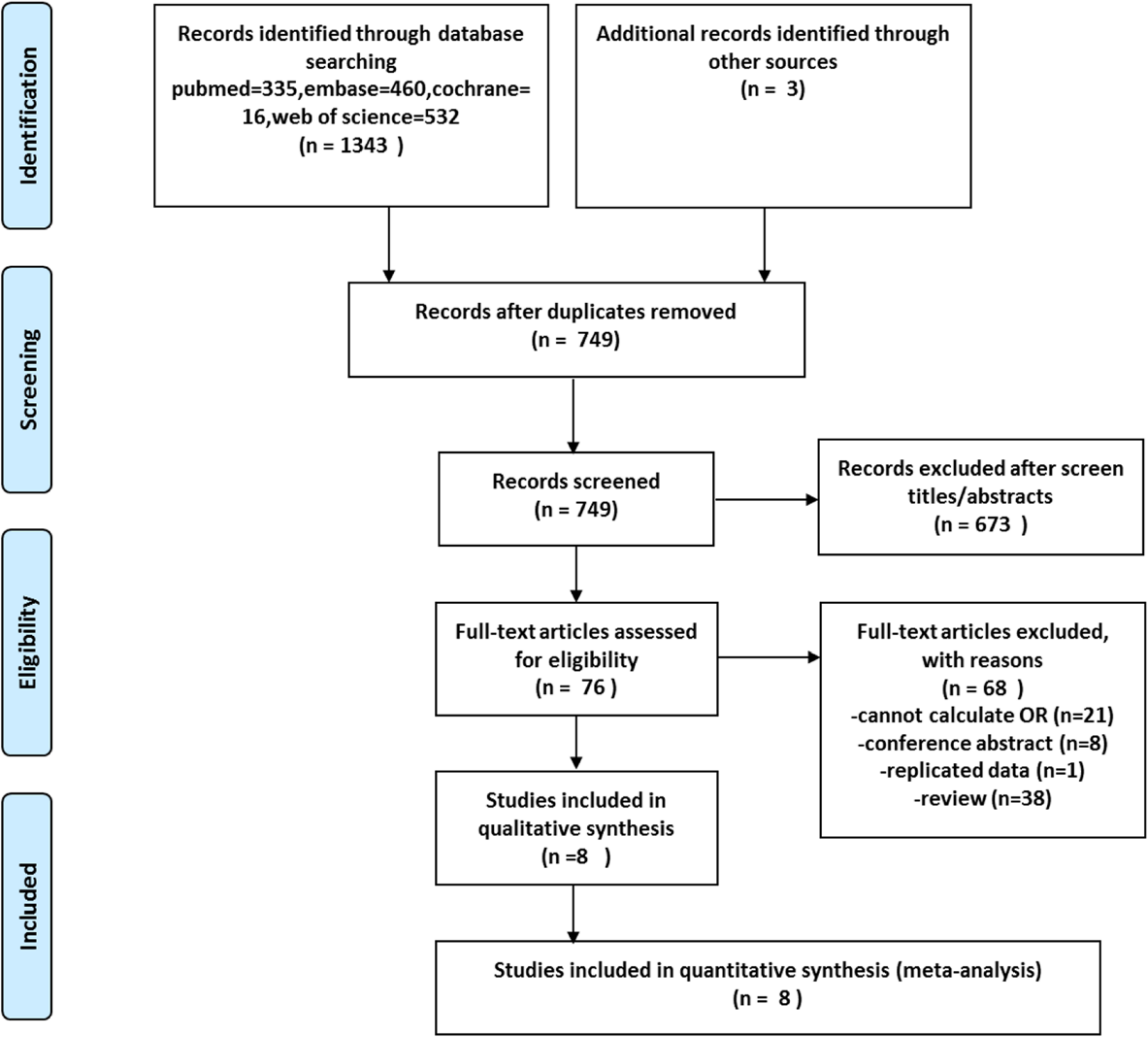
PRISMA [13] flow chart of literature review and data abstraction

The literature search yielded 749 records (include three studies from hand searching of references) after duplicates were removed. Of these, 673 records were excluded on the basis of their title and abstract. Review of the remaining 76 full articles led to 68 exclusions based on criteria described above. Finally, eight studies fulfilled our inclusion criteria and were included in our meta-analysis with a total number of 2462 PD patients and 3818 health controls (Fig. 1).

Of these, seven studies were case-control designs [14–20], and one was a cross-sectional study [21]. Most of the studies were conducted in Europe and North America [15, 16, 18–21], with one conducted in Asia [14], and one study conducted in Africa [17]. We divided the eight studies into two subgroups according to different RBD assessment methods described in the articles. If RBD was diagnosed with ICD (International classification of disease), RBD-SQ (RBD- screening questionnaire) or PSG, the symptom was defined as definite RBD [14, 16, 21]. If RBD was diagnosed with NMS-SQ, self-made Questionnaire or MSQ (Mayo Sleep Questionnaire), the symptom was defined as probable RBD [15, 17–20]. Summary characteristics for all included studies are provided in (Table 1). With NOS quality criteria, all studies scored ≥ 7/9 and six of the included studies scored 8/9.

**Table 1:** Characteristics of studies included in the Meta-analysis

Author	Year	Study design	Country	Population	PD cases	PD + RBD Number	Control Number	Con + RBD Number	RBD assessment	RBD diagnosis	RBD assessment before or after PD diagnosis	NOS score
Wu [14]	2015	case-control	Taiwan	Taiwan National Health Insurance Research Database	705	30	2820	33	ICD-9-CM	definite	before	8
Sunyer [15]	2015	case-control	Spanish and Austrian	outpatient clinics	109	30	107	3	NMS-SQ	probable	after	8
Baig [16]	2015	case-control	UK	Oxford Parkinson Disease Center	796	253	287	42	RBD-SQ	definite	after	8
Trinh [17]	2014	case-control	Tunisia	Institut National de Neurologie, Tunis	390	100	124	10	Questionnaire	probable	after	7
Doring [21]	2014	cross-section	Germany	Paracelsus Elena Klinik in Kassel	158	40	110	2	PSG	definite	after	8
Khoo [18]	2013	case-control	UK	Newcastle-upon-Tyne and Gateshead	159	55	99	8	NMS-SQ	probable	after	8
Adler [19]	2011	case-control	USA	Banner Sun Health Research Institute	49	34	175	23	MSQ	probable	after	8
Chaudhuri [20]	2006	case-control	Multicenter	Multicenter	123	40	96	10	NMS-SQ	probable	after	9
Total					2462	582 (23.6%)	3818	131 (3.4%)				

*PD* Parkinson disease, *RBD* Rapid eye movement behavior disorder, *ICD* International classification of disease, *NMS* non-motor symptom, *SQ* screening questionnaire, *MSQ* Mayo Sleep Questionnaire, *PSG* polysomnography, *NOS* Newcastle-Ottawa Scale

**Fig. 2:**
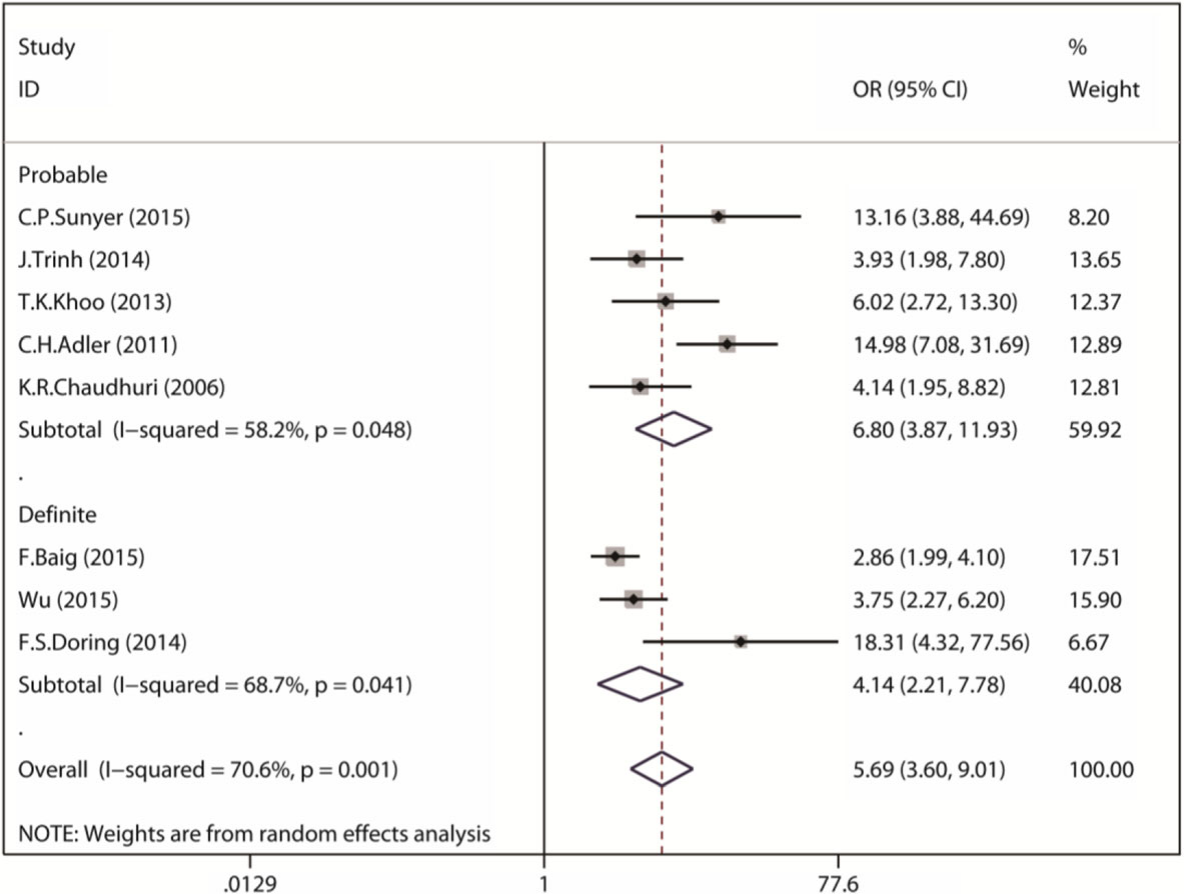
Forest plot demonstrating increased RBD risk in those with PD as compared with those control (CI, confidence interval; OR, odds ratio)

**Fig. 3:**
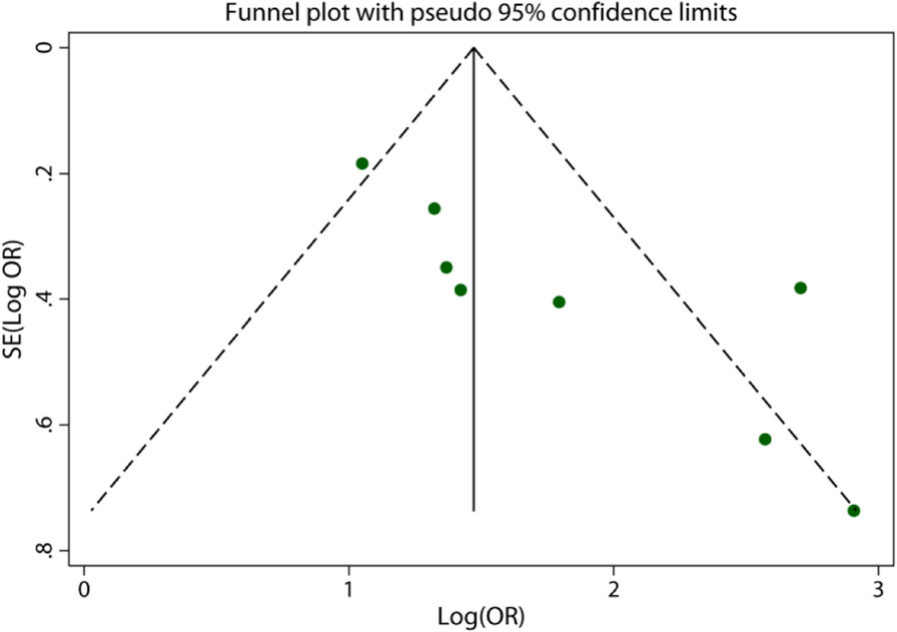
Funnel plot assessing publication bias in the study of RBD association with PD

The overall prevalence of RBD symptoms in PD was 582/2462 (23.6%) compared to 131/3818 (3.4%) in control. And the pooled OR was 5.69 (95% CI 3.60 to 9.01) with a moderate heterogeneity (I^2^ = 70.6%; *p* = 0.001) (Fig. 2). Subgroup analysis according to different diagnostic methods showed that the pooled OR for “Probable RBD” group was 6.08 (95% CI 3.87 to 11.93) with evidence of moderate heterogeneity (I^2^ = 58.2%; *p* = 0.048) and 4.14 for “Definite RBD” (95% CI 2.21 to 7.78) with medium heterogeneity (I^2^ = 68.7%; *p* = 0.041). After excluding the study of low weight [15, 19, 21], the overall polled OR was 3.54 (95% CI 2.77 to 4.52) and the heterogeneity was completely eliminated (I^2^ = 0%; *p* < 0.00001). Visual inspections of the funnel plot revealed a little asymmetry (Fig. 3) and significant publication bias was detected from results of the statistical test (Begg test: *p* = 0.019; Egger test: *p* = 0.011).

The results of our meta-analysis show a definite association between PD and RBD symptoms. Since studies included were of different study design, population bases and methods of exposure ascertainment, the heterogeneity across the studies was high. To identify studies that may have contributed to the pooled heterogeneity, we removed each of the eight studies (one study at a time) and examined the magnitude and direction of the polled relative risk (RR) after the removal of each study. This approach was repeated until the I^2^ statistic was deemed zero. Three studies [15, 19, 21] were identified as the source of heterogeneity in this study. After removing all of those studies, the polled OR was 3.54 (95% CI 2.77 to 4.52; I^2^ statistic = 0%), and the association between PD and the risk of developing RBD symptoms was still statistically significant.

## Discussion

Our systematic review and meta-analysis offers a confirmation of the association between PD and a subsequent diagnosis of RBD. The pooled prevalence of RBD symptoms in PD and control is 23.6 vs. 3.4%, and the patients who had symptoms of PD carried a 3.60 to 9.00-fold increased risk for developing RBD compared to health controls. It is important to quantify the magnitude of the association between PD and subsequent RBD symptoms, as this may underpin efforts to identify higher risk participants for entry to interventional studies with neuroprotective aims.

REM sleep behavior disorder (RBD) is a parasomnia disorder characterized by repeated episodes of dream enactment behavior and REM sleep without atonia (RSWA) during polysomnography (PSG) [22]. The main suspected mechanism of RBD is a lesion in the REM sleep atonia system, which is mainly located in the pontomedullary area [23]. RBD is important as a valuable preclinical marker of PD. Firstly, idiopathic RBD converted to neurodegenerative disease at a high rate ranging from 28 to 82% [24–27]. Secondly, many potential early marker of PD are significantly abnormal in iRBD, such as decreased striatal dopamine transporter binding, marked EEG slowing, substantial nigra hyperechogenicity and impaired olfaction [28–30]. Lastly, RBD is also associated with some specific PD phenotypes. It is known that age, gender, disease duration, motor disability, dopaminergic drug, motor phenotype, cognition, and autonomic dysfunction are different in PD patients with RBD compared to PD patients without the disorder [7].

Our study demonstrates that there is potential for publication bias based on the funnel plot and significant publication bias was detected from results of the statistical test (Begg test: *p* = 0.019; Egger test: *p* = 0.011). This might result in an overestimation of the association between PD and the risk of RBD symptoms. Considering that we only included eight studies which were assessed via means of the NOS, and all studies included in the main analysis had scores ≥ 7/9, we can conclude that the risk estimate that resulted from this analysis may also be viewed as a fairly strict estimate, and a result of the pooling of data from only the highest quality studies.

Similar to any other meta-analysis of observational studies, our study is subject to several limitations. First, we limited our search to publications in English and therefore might have missed a small number of relevant publications. Second, recall bias may affect the quality of information retrieved from participants in case-control studies and the assessment of RBD symptoms may vary from one study to another. Third, more high quality and large sample sizes observation studies estimating the risk of RBD symptoms in PD should be included in order to avert the publication bias.

## Conclusion

In conclusion, despite these limitations, we pooled data from 6280 people across eight studies to provide a consolidated risk that estimated relating PD to the development of RBD symptoms. Most of the evidence was of the highest quality and the conclusion was considered robust. Further prospective research is needed to examine the presence of RBD symptoms and its relationship to PD, in order to better understand the etiology and natural history of PD.

## Abbreviations

MSQ: Mayo sleep questionnaire
NMS: Non-motor symptoms
NOS: Newcastle-Ottawa Scale
OR: Odds ratio
PD: Parkinson’s disease
PSG: Polysomnography
RBD: Rapid eye movement behavior disorder
RSWA: REM sleep without atonia
